# Characteristics and general practice resource use of people with comorbid cancer and dementia in England: a retrospective cross-sectional study

**DOI:** 10.1186/s12875-022-01882-w

**Published:** 2022-11-12

**Authors:** Michelle Collinson, Ellen Mason, Rachael Kelley, Alys Griffiths, Laura Ashley, Ann Henry, Hayley Inman, Fiona Cowdell, June Hennell, Liz Jones, Maria Walsh, Margaret Ogden, Amanda Farrin, Claire Surr

**Affiliations:** 1 Clinical Trials Research Unit, Institute of Clinical Trials Research, University of Leeds, Leeds, LS2 9JT UK; 2 Centre for Dementia Research, School of Health and Community Studies, Leeds Beckett University, Leeds, UK; 3 Leeds School of Social Sciences, Leeds Beckett University, Leeds, UK; 4 Clinical Oncology, Leeds Teaching Hospitals NHS Trust, Leeds, UK; 5 School of Medicine, University of Leeds, Leeds, UK; 6 Clinical Oncology, Bradford Teaching Hospitals NHS Foundation Trust, Bradford, UK; 7 Faculty of Health, Education and Life Sciences, Birmingham City University, Birmingham, UK; 8 Patient and Public Involvement Representative, Leeds, UK

**Keywords:** Cancer, Dementia, Older adults, Primary health care, Administrative data, Electronic health records, Health care resource use, Cross-sectional study

## Abstract

**Background:**

Cancer and dementia are common in older people and management of the conditions as comorbidities can be challenging, yet little is known about the size or characteristics of this group. We aimed to estimate the prevalence, characteristics and general practice resource usage of people living with both conditions in England.

**Methods:**

Anonymised electronic healthcare records from 391 National Health Service general practices across England using the TPP SystmOne general practice system were obtained from ResearchOne. Data included demographic and clinical characteristics, and general practice healthcare useage (appointments, prescriptions, referrals and secondary care contacts) for people aged 50 and over with a cancer and/or dementia diagnosis consistent with the Quality and Outcomes Framework between 2005 and 2016. Multi-level negative binomial regression was used to analyse the association between having cancer and/or dementia and the number of general practice appointments.

**Results:**

Data from 162,371 people with cancer and/or dementia were analysed; 3616 (2.2%) people were identified as having comorbid cancer and dementia. Of people with cancer, 3.1% also had dementia, rising to 7.5% (1 in 13 people) in those aged 75 and over. Fewer people with both conditions were female (50.7%) compared to those with dementia alone (65.6%) and those with comorbid cancer and dementia were older than those with cancer alone [mean ages 83 (sd = 7), 69 (sd = 12) respectively]. Those with both conditions were less likely to have lung cancer than those with cancer alone (7.5% vs. 10.3%) but more likely to have prostate cancer (20.9% vs. 15.8%). Additional comorbidities were more prevalent for those with both conditions than those with cancer or dementia alone (68.4% vs. 50.2% vs. 54.0%). In the year following the first record of either condition, people with cancer and dementia had 9% more general practice appointments (IRR:1.09, 95% CI:1.01–1.17) than those with cancer alone and 37% more appointments than those with dementia alone (IRR: 1.37, 95% CI: 1.28–1.47).

**Conclusions:**

A significant number of people are living with comorbid cancer and dementia in England. This group have additional comorbidity and higher general practice usage than those with cancer/dementia alone. The needs of this group should be considered in future general practice care planning and research.

**Supplementary Information:**

The online version contains supplementary material available at 10.1186/s12875-022-01882-w.

## Background

Co-morbidity, the simultaneous presence of two chronic health conditions [1], is one of the greatest challenges facing healthcare services in high income countries [2]. It is estimated that 23.2% of the UK population live with comorbidity, with prevalence rising to 64.9% in those aged 65 and over and 81.5% in those aged 85 and over [3]. By 2035 it is estimated that additional gains in life expectancy will lead to many individuals living these extra years of life with four or more comorbid conditions (men 65.9%, women 85.2%) [4]. Comorbidity complicates clinical management and is associated with increased health resource use and worse health outcomes [1, 5].

Cancer and dementia are two highly prevalent conditions in older people and thus are common as comorbid conditions. Cancer as a comorbid condition is predicted to see one of the biggest increases in prevalence by 2035 (23.7% of those aged 65 and above, an increase of 179.4% from 2015 to 2035). For two-thirds of those aged 65+ living with four or more co-morbid conditions, one will be a condition that affects their mental or neurological health such as dementia, other types of cognitive impairment or depression [4]. Despite high prevalence rates in the over 65 s and the management complexities that comorbidity brings to both conditions, relatively little is known about comorbid cancer and dementia (CCD). We define CCD as a person with a cancer diagnosis who is also living with a diagnosis of dementia, irrespective of whether the cancer or dementia diagnosis was received first. This reflects the existing CCD literature which does not differentiate between people based on which diagnosis was received first, or on the basis of either dementia or cancer representing the specific index condition that is a feature of some definitions of comorbidity [1].

A 2018 systematic review [6] identified 34 studies all of which adopted different methods for estimating cancer and dementia comorbidity rates, leading to wide variation of prevalence rate estimates (0.2 to 45.6%). Thirty-one of the studies examined prevalence of dementia in samples of people with cancer. These studies were heterogeneous with regard to the cancer type(s) included (e.g. single or multiple), sampling (cancer in people with dementia or dementia in people with cancer), methods of identifying cancer and/or dementia within healthcare records, location (national, regional, local, individual hospital or nursing home databased), and setting (e.g. cancer registry, medical centre, long-term care or hospice data), meaning it was not possible within the review to draw robust conclusions regarding the prevalence of CCD. Most studies were conducted in the USA and Denmark, none were UK-based and only seven covered multiple cancer types. Despite more recent publication of studies that explored cancer/dementia comorbidity prevalence in certain cancer types or following a cancer diagnosis or receipt of certain cancer treatments see for example [7–11], relatively little remains known about the size and characteristics of the population of people with CCD. Our study addresses this gap by using data from primary care to identify people in England recorded as having a concurrent cancer (any type) and dementia diagnosis, thus drawing from a wider sample than previous studies.

The limited research on the treatment and support of people with CCD focusses predominantly on cancer diagnosis, treatment and care within specialist oncology services. This literature identifies that people with an existing dementia are diagnosed with cancer at a later stage, and those with CCD are less likely to receive cancer treatment and more likely to experience treatment complications than individuals with cancer alone and are at increased risk of death compared to people with other cancer comorbidities [6, 12].

Evidence indicates management of comorbidities in people with cancer is largely undertaken in primary care, with General Practitioners more likely to see holistic management of the patient as part of their role than oncology specialists [13]. A study of primary care use in people with cancer in their final year of life [14] found most were aged 70 years or older, had complex care needs, and the majority (75.1%) had comorbidities. The latter being associated with greater service use, more prescriptions and total number of medications prescribed. A Canadian study of older adults recently diagnosed with cancer [15], found similar high primary care service use, alongside high rates of emergency department use and hospitalisations. This study adds to our understanding of primary care use by people with comorbidities.

These findings in relation to cancer and comorbidity are particularly relevant when considering dementia as a comorbid condition. A large, cross-sectional analysis of primary care data concluded that people with dementia have higher rates of comorbidity than those without, with 19.0% reported to have 5 or more comorbid conditions [16]. Higher rates of comorbid conditions alongside dementia are associated with significantly higher rates of primary care consultations, prescriptions, hospitalisations and increased risk of death [17]. Evidence across a range of prevalent comorbidities indicates people with dementia are less likely to receive the same quality of care or access to services for their comorbid condition than those without dementia [18]. This suggests those with CCD may be more likely to have other comorbidities, may be more likely to be accessing primary care for management of the conditions and symptom management for this group may be more complex. However, there is scarce literature on health resource use or care complexity in people with CCD.

Dementia adds additional complexity to treatment and management of cancer for clinicians, patients and their families [19]. Given there is less active treatment of people with CCD in oncology services [6], their healthcare needs may be met by other services, or may be under-managed. The only published study to date examining health resource use in people with CCD [20] compared the health resource use of 96,124 Medicare beneficiaries aged 65 and over with cancer, dementia, CCD and neither cancer or dementia, in one region of the USA. They found that people with CCD were most likely to have additional comorbidities (81.1%) followed by those with cancer alone (58.3%), those with dementia alone (52.4%) and those with neither dementia nor cancer (21.7%). Individuals with CCD had the highest rate of hospitalisations (30.7%), 30-day readmissions (23.5%), emergency department use (74.3%), and intensive care unit use (38.9%), followed by those with dementia alone and then those with cancer alone. Those with CCD had the highest number of visits with their physician (mean = 5.6, sd = 6.4), and the greatest proportion of patients with 6 or more physician visits over the 12-month period (37.3%), again followed by individuals with dementia alone, than cancer alone. Those with CCD and dementia alone had similar proportions of the cohort who had ever resided in a nursing home (48.5% and 49.6% respectively). The authors concluded that people with CCD present higher health services costs than other groups, indicating the need to develop co-ordinated, comprehensive care plans for this population that consider their holistic healthcare needs. To date there have been no UK studies examining health resource use of those with CCD compared to those with only one of the conditions or none, and it is unclear whether a similar picture of high healthcare resource use alongside poorer health outcomes exists.

Electronic health records (EHRs) are a key feature of English general practice and almost every primary care consultation is recorded [21]. Primary care EHRs are recognised as the most accurate routinely collected dataset available for identification of all-cause dementia in the UK [22] as there is currently no national dementia registry. In addition, records include indicators across clinical areas (including cancer and dementia) relevant to the Quality and Outcomes Framework (QOF), a voluntary annual scheme that rewards general practices (GP) in England for achievement of indicators of good practice via incentive payments [23]. This study aimed to use data from GP based primary care to estimate the size of the population of people with CCD in England and to describe their socio-demographic and general practice service-use characteristics, compared to those with dementia alone, cancer alone and an aged-matched cohort of the general population.

## Methods

Methods and results are reported in accordance with STROBE guidelines.

### Study design and Setting

A cross-sectional study was conducted using anonymised EHRs from 391 National Health Service GPs across England (5.1% of practices) who were using the TPP SystmOne [24] GP system. Data were obtained via ResearchOne [25], a health and care research database in February 2018.

#### Participants

Records for 162,371 patients aged 50 and over with a QOF registered diagnosis of cancer and/or dementia between 2005 and 2016 were available. This age group was intentionally chosen as it is from age 50 when cancer and dementia prevalence / incidence rates increase. Routinely collected data on demographic and clinical characteristics, appointments, prescriptions, referrals and secondary care contacts were available. Aggregate level comparator data for demographic variables was provided by ResearchOne for the whole population aged 50 and over (as of 02/03/2017) present in the ResearchOne database. Comparator general population summaries of general practice appointments, prescriptions and referrals were provided for the 1-year period 01/01/2015 to 01/01/2016. A population estimate of 20,399,661 people aged 50 and over in England in 2017 was obtained from the Office for National Statistics [26] to aid the prevalence calculation.

#### Variables

The populations of interest for this study were those with 1) comorbid cancer and dementia, 2) cancer alone, 3) dementia alone and 4) those in the comparator group provided by ResearchOne.

The outcomes for this study were defined as:

- Prevalence of comorbid cancer and dementia
- Descriptive summaries of demographic and clinical characteristics for each population
- Descriptive summaries of GP based primary healthcare useage for each population
- Descriptive summaries of secondary healthcare useage for each population
- Differences in demographic (gender, age, ethnicity, region, IMD, living in a care home, number of comorbidities, deaths within 12 months), clinical (cancer type, dementia type) and healthcare usage between the populations

#### Data sources and measurement

The first QOF record of cancer and/or dementia in the EHRs was identified for each patient. To aid analysis and interpretation, data were categorised using CTV3 Read Codes (a coded thesaurus of clinical terms used in the National Health Service to categorise medical and administrative activity in patient EHRs [27]). Records were reviewed by the research team and categorised into the UK’s top 20 most common cancers [28] and 8 most common dementia types [29].

A patient was defined as having CCD if: (1) a QOF record of dementia occurred prior to a QOF record of cancer; OR (2) a QOF record of cancer occurred in the 5 years prior to a QOF record of dementia; OR (3) a QOF record of cancer and dementia occurred on the same date. Five-years was chosen as it is the most commonly used period of cancer survivorship/remission during which cancer is likely to return, with remission beyond this period deemed to reflect ‘lack of cancer’ [30].

Ethnicity was grouped into standardised major ethnic group categories [31]; Index of Multiple Deprivation (IMD) rank score (a measure of deprivation) was grouped into deciles [32] and middle layer super output area was mapped to 14 UK geographical regions [33, 34] (see Supplementary Table S1). Care home residence was predetermined by ResearchOne by matching patient postcodes to those of Care Quality Commission (independent regulator of health and social care in England) registered care homes. CTV3 Read Codes for long-term health conditions (QOF registered) were categorised into 28 conditions by ResearchOne and to aid comparison with other research, these were further categorised by the research team into 22 long-term conditions (e.g. Type 1 and Type 2 diabetes were combined into a ‘Diabetes’ category). Patient age, IMD, region of residence and care home status at the time closest to the first QOF record of either cancer, dementia or the point a patient had both conditions was used for analysis.

General practice appointments were defined as those having taken place in the year following the first QOF record of cancer and/or dementia. For example, if the first QOF record of dementia was 01/01/2010 then any general practice appointments occurring between 01/01/2010 and 01/01/2011 were included. GP appointments included appointments with General Practitioners, nurses and other health professionals (e.g., clinical psychologist, dietician, occupational therapist). Prescriptions and referrals from general practice and secondary care contacts (Accident & Emergency department attendances, hospital admissions and outpatient appointments) were those recorded in EHRs within the same one-year time period.

#### Study size and Bias

The study size was restricted by the availability of data within the ResearchOne database. Records for 162,371 patients were available for analysis. Completeness of data within the ResearchOne datasets was dependent on the completeness of the electronic records in SystmOne. Missing data may therefore have resulted in patients being incorrectly included or excluded from the populations of interest however it was not possible for us to explore this within the datasets provided.

#### Quantitative variables

A prevalence estimate for the number of people with cancer and dementia was calculated by dividing the number of people with cancer and dementia in our sample by the number of people aged 50 and over in England in 2017, as obtained from ONS data and multiplying by 1000. Demographic and clinical characteristics (age, gender, ethnicity, IMD, care home residence, region of residence, cancer and/or dementia type, comorbidities) were summarised for people with cancer only, dementia only and comorbid cancer and dementia. The number of general practice appointments per patient per month within the first year following the first QOF record of cancer and/or dementia were summarised descriptively, taking into account the number of months the patient was alive. This was repeated for numbers of referrals, medications and secondary care contacts. Where possible, the extent of missing data was summarised.

#### Statistical analysis

To account for patients clustered within general practices, multi-level negative binomial regression [35] was used to investigate the association between having cancer and/or dementia and the number of general practice appointments. The model adjusted for patient level covariates only (age, gender, IMD, ethnicity, number of comorbidities and geographical region), as no general practice level covariates were available in the data. An offset variable was included to account for the number of months alive within the time period. Adjusted incidence rate ratios (IRRs), 95% confidence intervals (CIs) and *p*-values were reported. Due to limitations with the data, statistical modelling of secondary care data was not conducted. All analyses were performed using SAS v9.4 [36].

## Results

### Participants

Data for 162,371 people aged 50 and over were included in the analysis; 112,772 people with cancer, 45,983 people with dementia and 3616 people with comorbid cancer and dementia.

### Prevalence of CCD

The prevalence of CCD in people aged 50 and over was 0.18 per 1000 people in England in 2017. In people aged 50 and over with dementia, 7.3% also had cancer, whilst 3.1% of people with cancer also had dementia. Of people aged 75 and over with cancer and/or dementia, 7.5% (1 in 13 people) had both conditions.

### Demographics

Demographic characteristics are shown in Table 1. Approximately half of those with CCD, those with cancer alone and the general population were female, compared to 65.6% of those with dementia alone. People with CCD were on average older than the cancer alone and general populations [mean ages 83 (sd = 7), 69 (sd = 12), 66 (sd = 11) respectively] and similar in age to the dementia alone population [mean age 82 (sd = 8)]. Prostate and breast cancers were most prevalent for those with CCD and cancer alone, although higher rates of both were noted in the CCD population compared to those with cancer alone (20.9% vs. 15.8% and 20.0% vs. 17.7% respectively). The distribution of dementia type was broadly similar across those with CCD and those with dementia alone, although a slightly lower proportion of the CCD group had unspecified dementia (42.7% vs. 45.5%), and a slightly higher percentage had vascular dementia (21.3% vs 19.2%). Ethnicity was comparable across all populations.

**Table 1:** Demographic characteristics of people with comorbid cancer and dementia compared to those with cancer or dementia alone and the general population aged 50 and over

Characteristic	Comorbid cancer and dementia (***n*** = 3616), n (%)	Cancer alone (***n*** = 112,772), n (%)	Dementia alone (***n*** = 45,983), n (%)	General population aged 50 and over^**a**^ (***n*** = 1,151,503), n (%)
Gender (% female)	1832 (50.7)	54,744 (48.5)	30,178 (65.6)	595,064 (51.7)
Age in years, mean (s.d.)	83 (7)	69 (12)	82 (8)	66 (11)
Age group				
< 50^b^	2 (0.1)	6672 (5.9)	117 (0.3)	0
50–74	477 (13.2)	67,318 (59.7)	7418 (16.1)	900,360 (78.2)
≥ 75	3137 (86.8)	38,782 (34.4)	38,448 (83.6)	251,143 (21.8)
Cancer type				
Other	1447 (40.0)	50,614 (44.9)	–	–
Prostate	757 (20.9)	17,799 (15.8)	–	–
Breast	724 (20.0)	19,974 (17.7)	–	–
Bowel	415 (11.5)	12,747 (11.3)	–	–
Lung	273 (7.5)	11,638 (10.3)	–	–
Dementia type				
Unspecified Dementia	1545 (42.7)	–	20,857 (45.4)	–
Alzheimer’s Disease	1093 (30.2)	–	13,458 (29.3)	–
Vascular Dementia	771 (21.3)	–	8836 (19.2)	–
Mixed Dementia	100 (2.8)	–	1260 (2.7)	–
Dementia with Lewy bodies	56 (1.5)	–	814 (1.8)	–
Frontotemporal Dementia	22 (0.6)	–	203 (0.4)	–
Parkinson’s Dementia	18 (0.5)	–	367 (0.8)	–
Other	11 (0.3)	–	188 (0.4)	–
Ethnicity				
White	2297 (63.5)	66,561 (59.0)	28,900 (62.8)	67.2
Not stated	343 (9.5)	8891 (7.9)	3865 (8.4)	6.0
Asian	30 (0.8)	1344 (1.2)	669 (1.5)	3.4
Black	17 (0.5)	475 (0.4)	200 (0.4)	0.9
Other	18 (0.5)	390 (0.3)	205 (0.4)	0.5
Mixed	4 (0.1)	186 (0.2)	80 (0.2)	0.2
Missing	907 (25.1)	34,925 (31.0)	12,064 (26.2)	21.7
Region				
Yorkshire and the Humber	1217 (33.7)	33,393 (29.6)	14,690 (31.9)	29.0
East of England	730 (20.2)	24,226 (21.5)	9334 (20.3)	21.8
East Midlands	546 (15.1)	19,189 (17.0)	7254 (15.8)	17.5
South West	518 (14.3)	17,055 (15.1)	6555 (14.3)	15.7
South East	279 (7.7)	9365 (8.3)	3956 (8.6)	8.7
North East	93 (2.6)	2956 (2.6)	1150 (2.5)	2.5
West Midlands	73 (2.0)	2251 (2.0)	714 (1.6)	1.9
North West	62 (1.7)	1583 (1.4)	707 (1.5)	1.3
London	51 (1.4)	1639 (1.5)	852 (1.9)	1.6
Outside of England^c^	1 (0.0)	150 (0.1)	66 (0.1)	0.0
Missing	46 (1.3)	965 (0.9)	705 (1.5)	0.1
IMD				
10% most deprived	314 (8.7)	8081 (7.2)	4280 (9.3)	7.5
10–20%	296 (8.2)	8184 (7.3)	3877 (8.4)	7.5
20–30%	298 (8.2)	8782 (7.8)	3738 (8.1)	7.9
30–40%	360 (10.0)	9803 (8.7)	4752 (10.3)	8.8
40–50%	403 (11.1)	11,984 (10.6)	5101 (11.1)	10.8
50–60%	453 (12.5)	13,927 (12.3)	6037 (13.1)	12.4
60–70%	354 (9.8)	11,744 (10.4)	4667 (10.1)	10.9
70–80%	368 (10.2)	12,306 (10.9)	4770 (10.4)	12.1
80–90%	353 (9.8)	12,236 (10.9)	3998 (8.7)	12.5
10% least deprived	217 (6.0)	8976 (8.0)	2653 (5.8)	9.7
Missing	200 (5.5)	6749 (6.0)	2110 (4.6)	0.0
Living in a care home	1582 (43.8)	5583 (5.0)	24,290 (52.8)	19,489 (1.7)
With comorbidity	2472 (68.4)	56,615 (50.2)	24,811 (54.0)	–
Number of additional comorbidities				
One	1111 (44.9)	32,064 (56.6)	12,803 (51.6)	–
Two	755 (30.5)	15,426 (27.2)	6920 (27.9)	–
Three	342 (13.8)	6172 (10.9)	3211 (12.9)	–
Four	166 (6.7)	2079 (3.7)	1244 (5.0)	–
Five	62 (2.5)	633 (1.1)	471 (1.9)	–
> Five	36 (1.5)	241 (0.4)	162 (0.6)	–
Died within 12 months^d^	1140 (31.5)	25,538 (22.6)	7579 (16.5)	–

^a^The general population includes those identified as having cancer or dementia

^**b**^Aged 50 or over at the date of data extraction, but the patient was aged less than 50 at the first QOF record of cancer and/or dementia

^**c**^People recorded as “outside of England” had a general practice appointment in England but their registered home address was outside of England (Wales, Scotland, Isle of Man, Channel Islands and Northern Ireland)

^d^Deaths recorded within the first year following cancer or dementia diagnosis or from when the CCD comorbidity was recorded (unadjusted)

### Area and type of residence

The majority of patients resided in Yorkshire and the Humber, East of England, East Midlands and the South West of England with comparable proportions across all populations. More people with dementia alone lived in one of the most deprived areas compared to people with cancer alone or those with CCD (9.3% vs. 7.2% vs. 8.7%). While few people in the general population (1.7%) and those with cancer alone (5.0%) were living in a care home, as expected, much higher proportions of people with CCD (43.8%) and dementia alone (52.8%) were living in care homes at the time closest to their cancer or dementia diagnosis or when their CCD comorbidity was recorded.

### Comorbidities

Those with CCD were more likely to have additional comorbidities (68.4%) compared to those with cancer (50.2%) or dementia (54.0%) alone. Of those who had at least one comorbidity, people with CCD were more likely (55.1%) to have two or more additional comorbidities (i.e. a minimum of four comorbid conditions) than the comparator groups (48.4% dementia alone and 43.4% cancer alone). A greater proportion of people with cancer alone had a single additional comorbidity, than those with dementia alone or CCD (56.6% vs. 51.6% vs. 44.9%).

### Deaths

People with CCD were more likely to die within the first year following the record of their CCD comorbidity (31.5%) compared to those within the first year of their cancer (22.6%) or dementia diagnosis (16.5%) as a single condition.

### General practice appointments

Data were available for 1,303,765 general practice appointments in the year period of analysis for each patient (i.e. in the year following their first QOF record of cancer or dementia or CCD). Those with CCD were more likely to have a GP based primary care appointment (59.2%) compared to those with cancer (51.4%) or dementia (54.6%) alone (see Table 2). All three groups were less likely to have a GP based primary care appointment than the general population (81.9%).

**Table 2:** Healthcare resource use of people with comorbid cancer and dementia compared to those with cancer or dementia alone and the general population aged 50 and over

	Comorbid cancer and dementia (***n*** = 3616), n (%)	Cancer alone (***n*** = 112,772), n (%)	Dementia alone (***n*** = 45,983), n (%)	General population aged 50 and over (***n*** = 1,151,503), n (%)
***General practice appointments***				
Number of appointments	29,879	761,894	298,343	8,520,666
Number of patients attending at least one appointment	2142 (59.2)	57,986 (51.4)	25,100 (54.6)	911,654 (81.9)
Number of appointments per person^a^				
Median (IQR)	9 (4,18)	10 (5,17)	7 (3,15)	–
Number of appointments per person per month^b^				
Mean (s.d.)	1.48 (2.18)	1.35 (1.80)	1.08 (1.69)	0.78 (0.79)
Median (range)	0.92 (0.08, 40.58)^e^	0.92 (0.08, 97.75)^e^	0.67 (0.08, 47.50)^e^	0.58 (26.75)^f^
***Prescriptions***				
Number of prescriptions	51,013	858,055	657,471	43,237,123
Number of patients with at least one prescription	2703 (74.8)	64,261 (57.0)	33,725 (73.3)	940,122 (81.6)
Number of prescriptions per person^a^				
Median (IQR)	13 (6, 25)	8 (3, 17)	14 (7, 26)	–
Number of items prescribed per person per month^b^				
Mean (s.d.)	1.97 (2.07)	1.48 (1.73)	1.75 (1.72)	3.83 (5.51)
Median (range)	1.33 (0.08, 32.67)	0.92 (0.08, 37.33)	1.25 (0.08, 30.25)	2.25 (254.75)^d^
***Secondary care contacts***^c^				
Number of contacts	2013	53,605	19,958	–
Number of patients with at least one contact	890 (24.6)	24,367 (21.6)	9490 (20.6)	–
Number of contacts per person^a^				
Median (IQR)	1 (1, 3)	1 (1, 2)	1 (1, 2)	–
Number of contacts per person per month^b^				
Mean (s.d.)	0.26 (0.29)	0.26 (0.32)	0.22 (0.24)	–
Median (range)	0.17 (0.08, 2.33)	0.17 (0.08, 6.00)	0.17 (0.08, 6.00)	–
*A&E Attendances*				
Number of contacts (% of contacts)	1028 (51.1)	18,054 (33.7)	11,515 (57.7)	–
Number of patients with at least one contact	608 (16.8)	11,926 (10.6)	6810 (14.8)	–
Number of contacts per person^a^				
Median (IQR)	1 (1, 2)	1 (1, 2)	1 (1, 2)	–
Number of contacts per person per month^b^				
Mean (s.d.)	0.20 (0.19)	0.18 (0.19)	0.17 (0.16)	–
Median (range)	0.13 (0.08, 2.00)	0.08 (0.08, 4.17)	0.08 (0.08, 3.00)	–
*Hospital Admissions*				
Number of contacts (% of contacts)	767 (38.1)	23,804 (44.4)	6988 (35.0)	–
Number of patients with at least one contact	432 (11.9)	12,893 (11.4)	4472 (9.7)	–
Number of contacts per person^a^				
Median (IQR)	1 (1, 2)	1 (1, 2)	1 (1, 2)	–
Number of contacts per person per month^b^				
Mean (s.d.)	0.20 (0.19)	0.22 (0.27)	0.17 (0.19)	–
Median (range)	0.14 (0.08, 1.00)	0.14 (0.08, 4.50)	0.08 (0.08, 6.00)	–
*Outpatient Appointments*				
Number of contacts (% of contacts)	211 (10.5)	11,548 (21.5)	1401 (7.0)	–
Number of patients with at least one contact	119 (3.3)	5066 (4.5)	848 (1.8)	–
Number of contacts per person^a^				
Median (IQR)	1 (1, 2)	1 (1, 2)	1 (1, 2)	–
Number of contacts per person per month^b^				
Mean (s.d.)	0.20 (0.28)	0.23 (0.31)	0.16 (0.14)	–
Median (range)	0.08 (0.08, 2.33)	0.08 (0.08, 4.00)	0.08 (0.08, 1.20)	–
*Unable to classify*^d^				
Number of contacts (% of contacts)	7 (0.3)	199 (0.4)	54 (0.3)	–
Number of patients with at least one contact	7 (0.2)	172 (0.2)	51 (0.1)	–
Number of contacts per person^a^				
Median (IQR)	1 (1, 1)	1 (1, 1)	1 (1, 1)	–
Number of contacts per person per month^b^				
Mean (s.d.)	0.09 (0.01)	0.16 (0.16)	0.11 (0.04)	–
Median (range)	0.08 (0.08, 0.11)	0.08 (0.08, 1.00)	0.08 (0.08, 0.25)	–

^a^Unadjusted for time alive

^b^For those patients attending at least one appointment / with at least one prescription / with at least one contact, adjusted for time alive

^c^Data on secondary care contacts for the general population were not provided by ResearchOne

^d^Records that indicate a secondary care appointment but the categorisation of whether this was an admission/A&E attendance/outpatient appointment is unclear e.g. hospital-based dietitian, hospital-based physiotherapist

^e^Includes all appointments that met our definition of an appointment, without the exclusion of outliers

^f^Minimum and maximum values of the range were not available from ResearchOne

Of patients that attended a GP based primary care appointment, people with CCD had a higher mean number of appointments per month across the year (1.48, sd = 2.18), compared to those with cancer alone (1.35, sd = 1.8), dementia alone (1.08, sd = 1.69) and the general population (0.78, sd = 0.79). People with CCD had 9% more GP based primary care appointments compared to those with cancer alone (IRR = 1.09; 95% CI = 1.01–1.17) and 37% more appointments than those with dementia alone (IRR = 1.37; 95% CI = 1.28–1.47). People with dementia alone had 21% less appointments compared to people with cancer alone (IRR = 0.79; 95% CI = 0.77–0.82) (see Table 3).

**Table 3:** Multivariate negative binomial models for general practice appointments

	Adjusted IRR^a^ (95% CI)		
Variables	Comorbid Cancer and Dementia^b^	Comorbid Cancer and Dementia^c^	Dementia Only^b^
Number of General Practice Appointments	1.09 (1.01–1.67)*	1.37 (1.28–1.47)**	0.79 (0.77–0.82)**

*IRR* Incidence Rate Ratio, *CI* Confidence Interval

^a^Models were adjusted for age, gender, ethnicity, IMD, count of comorbidities and region

^b^Reference group is “Cancer only”

^c^Reference group is “Dementia only”

* *p* < 0.05, ** *p* < 0.001

### Prescribed medications

A higher proportion of the general population had at least one item prescribed by general practice within the one-year time period (81.6%) compared to people with CCD (74.8%), dementia alone (73.3%) and cancer alone (57.0%). The general population also had a higher mean number of items prescribed per person per month (3.83, sd = 5.51) compared to those with CCD (1.97, sd = 2.07), with cancer (1.48, sd = 1.73), or dementia (1.75, sd = 1.72).

### Secondary care contacts

The proportion of people with at least one recorded secondary care contact was slightly higher for those with CCD (24.6%) than compared to people with cancer alone (21.6%) and dementia alone (20.6%), although the mean number of contacts per person per month was similar across the populations. More of the contacts that people with dementia alone had were Accident & Emergency department attendances than those with cancer alone or those with CCD (57.7%, 33.7%, 51.1%). More hospital admissions (44.4%, 38.1%, 35.0%) and outpatient appointments (21.5%, 10.5%, 7.0%) were noted for people with cancer alone than people with CCD or dementia alone.

## Discussion

This is the first study to use an English GP based primary care dataset to estimate the size, characteristics and health resource use of people aged 50 and over with CCD (including all types of dementia and cancer) and to compare this group to cohorts with cancer alone, dementia alone and the general population of this age group. Our data provides the first robust estimates of the prevalence of CCD in England, indicating a rate of 0.18 per 1000 in those aged 50+. We have shown that in those aged 75 and over with cancer or dementia, 1 in 13 (7.5%) live with both conditions. Those with CCD, compared to those with cancer or dementia alone, are older on average (83 years), more likely to have additional comorbidities (68.4%), have higher mortality in the first year after diagnosis (31.5%) and have higher health resource including prescription medications (74.8%), and use of primary (59.2%) and secondary care services (24.6%).

Previous studies have attempted to assess the size of the population of people with CCD, producing wide-ranging estimates [6]. The proportion of people diagnosed with dementia in England doubled over the period our data covers [37] but as, at most two thirds of those with dementia have a formal diagnosis [38], our figures of CCD prevalence are likely to under estimate the size of this population. Our study highlights the inaccuracies of previous methods used to estimate the size of this population and the need to develop and utilise robust datasets, in order to systematically examine prevalence rates and describe population demographics.

Our findings are broadly consistent with the only other study to date exploring the demographics and healthcare resource use of people with CCD compared to those with cancer or dementia alone. Similarly to Kedia et al [20]. we found people with CCD in England were more likely to have additional comorbidities (68.4%) than those with dementia alone or cancer alone. While the percentage of the population with additional comorbidities was lower in our sample (68.4% vs 81.1%) this is likely to result from the older population sampled in the US study (65 years and over vs. 50 years and over). It has been noted that interventions for older people having cancer treatment rarely target or address comorbidity [39]. Given the complexity that comorbidity adds to cancer treatment [40] and the greater prevalence of additional comorbid conditions in patients who already have both cancer and dementia, this is an area that warrants further investigation. In particular, the impact of multiple comorbid conditions on ongoing care, such as treatment decision-making and symptom management, should be considered.

The higher average rates of GP based primary care visits in those with CCD in our sample compared to the other groups (59.2% CCD, 54.6% dementia alone and 51.4% cancer alone) may be explained by their higher rates of additional comorbidities. Greater primary care use in people with CCD may therefore be a positive indicator of needs appropriate access to healthcare services. While primary care resource use is reported to be high in the final year of life for those with cancer [14], people with dementia often access less community-based care than their older counterparts, despite greater healthcare needs [41] and are thus high users of emergency departments [42]. Emergency department use is also high in people with cancer [43, 44]. In Kedia et al.’s US study, people with CCD were the highest users of emergency department services, as might be predicted by a cumulative effect of this comorbidity. However, our data did not replicate this finding, with people with dementia alone having more emergency department attendances (57.7%), than those with CCD (51.1%) or cancer alone (33.7%). The reason for this is unclear, although it may be that the higher rates of GP-based primary care and secondary care in our CCD sample are indicative of accessing timely and proactive healthcare thus reducing the need for emergency care. Emergency department use in people with dementia frequently results in hospitalisation [45], often for conditions that could potentially be managed at home [41]. Increased risk of hospitalisation in people with dementia is associated with older age, multi-morbidity and polypharmacy [46], all of which were higher in our CCD group. It is therefore perhaps unsurprising that people with CCD in our study accessed hospital secondary care services more often than the other groups, a finding also reported in Kedia et al’s US study [20]. Our data was not able to examine the reason for different healthcare contacts and type of secondary care contact (e.g. outpatient, admission etc.). Healthcare contacts for people with CCD are complex and inter-related and future research is warranted to explore the reasons for contacts with different healthcare services and appropriateness of these with a view to reducing unnecessary emergency department use and hospital admissions where appropriate.

Our study showed 43.8% of people with CCD and 52.8% of those with dementia alone were living in a care home at the time of/closest to their diagnosis. A similar picture was found by Kedia et al. in the US [20]. These figures indicate a significant number of people with CCD are cared for in a long-term care facility at some point in their illness trajectory. However, little research has been conducted on CCD and its management in long-term care, with existing studies addressing prescription of analgesia and presence of behavioural and sleep disturbances [47, 48] and end-of-life care management [49]. These studies highlight the additional complexities CCD brings to resident management and care. Further research on the care and management of residents with CCD in long-term care facilities and the support health and social care staff may need to deliver this is warranted, given the likely size of this population alongside their complex needs.

We can conclude that people with CCD present higher health services use and thus costs than other groups, a finding in line with those found in a US context by Kedia et al. [20]. What we cannot extrapolate from our data are the reasons for this. Further research to explore reasons for access and outcomes for people with CCD across the healthcare system, to identify areas for potential intervention is needed. Our study also indicates that there are potentially many older people with CCD using primary and secondary care services and residing in long-term care. Little is known about the specific management, care and support needs of this population and there is little or no guidance for clinicians to draw on in planning and managing their care. Oncology staff may have had little opportunity to access dementia training and may feel poorly equipped to support dementia-specific or related needs [19]. Likewise, primary care practitioners often feel they would benefit from further training and clinical guidelines on managing cancer [50, 51] and dementia [52, 53] in primary care. Improved training and development of clinical guidance on management of CCD alongside better integration of primary and secondary care [54] are required.

### Strengths and limitations

The size of the sample is a key strength of our study; we included 112,772 people with cancer, 45,983 with dementia and 3616 people with comorbid cancer and dementia. It is the first in the UK to use a large routine dataset to examine the size, characteristics and health resource use of this population. Several limitations should be noted. Our sample over represents general practices in specific areas of England, and the sample may not be representative of the country as a whole. No standard set of data dictionaries were available for our dataset and data quality was variable by general practice, and often included free-text options rather than fixed categories and had large amounts of missing data for some items (e.g. ethnicity). We were thus required to generate rules and assumptions, which may limit any conclusions drawn. Entry onto the QOF register cannot be assumed to correspond to time of diagnosis with a condition given amendments to QOF categories over time and dementia only being included as a condition in 2006/7. As the data relate to GP based primary care, data on secondary care contacts was limited, as this data is not always entered into general practice patient records in a way that allows easy extraction. Reasons provided for secondary care contacts were not comprehensive and summaries of secondary care data are likely to underestimate the true number and nature of contacts. Therefore, statistical modelling and comparison of this data to the general population provided by ResearchOne was not possible. Future research should consider linkage of GP based primary care data to alternative data sources such as Hospital Episode Statistics, to allow greater understanding of the secondary healthcare usage of people with cancer and/or dementia. We were unable to investigate other data items in detail (e.g. prescription medications), relying on crude counts. A small number of patients in our dataset (1.9%) had a disproportionately large number of recorded General Practitioner contacts, likewise the range of prescribed medications in the general population data provided by ResearchOne was large. These outliers may have skewed the mean/median for these outcomes. Due to the nature of the dataset, we were provided with the general population summaries by ResearchOne at the time of data release, rather than the raw dataset. Therefore, we were unable to further explore reasons for differences between the general population data and those of our specific sample groups (e.g. more General Practitioner attendances, higher rates of prescriptions medications etc.)

Our findings show that a substantial number of older people in England are living with both cancer and dementia and they have higher general practice resource usage than those with cancer or dementia alone. Whilst this is not entirely unexpected, this is one of the first studies to robustly quantify the size and characteristics of the population which can be used to help inform resource planning and justify future research to improve the care received by this group.

Future research should focus on exploring reasons for resource usage by people with CCD and include an in-depth examination of prescribing/dispensing and secondary care data in order to better understand whether, where and how more optimal care for people living with comorbid cancer and dementia could be provided.

## Supplementary Information

**Additional file 1: Table S1.** Data categories.

## Data Availability

The data that support the findings of this study are available from ResearchOne but restrictions apply to the availability of these data, which were used under license for the current study, and so are not publicly available. Data are however available from the authors (MC) upon reasonable request and with permission of ResearchOne.

## Abbreviations

CCD: Comorbid Cancer and Dementia
CI: Confidence Interval
EHR: Electronic Health Record
GP: General Practice
IMD: Index of Multiple Deprivation
IRR: Incidence Rate Ratio
ONS: Office for National Statistics
QOF: Quality and Outcomes Framework

## References

[1] Valderas JM, Starfield B, Sibbald B, Salisbury C, Roland M (2009). Defining comorbidity: implications for understanding health and health services. Ann Fam Med.

[2] Department of Health (2014). Comorbidities: a framework of principles for system-wide action.

[3] Barnett K, Mercer SW, Norbury M, Watt G, Wyke S, Guthrie B (2012). Epidemiology of multimorbidity and implications for health care, research, and medical education: a cross-sectional study. Lancet.

[4] Kingston A, Robinson L, Booth H, Knapp M, Jagger C, for the MODEM project (2018). Projections of multi-morbidity in the older population in England to 2035: estimates from the population ageing and care simulation (PACSim) model. Age Ageing.

[5] McPhail S (2016). Multimorbidity in chronic disease: impact on health care resources and costs. Risk Manag Healthcare Policy.

[6] McWilliams L, Farrell C, Grande G, Keady J, Swarbrick C, Yorke J (2018). A systematic review of the prevalence of comorbid cancer and dementia and its implications for cancer-related care. Aging Ment Health.

[7] Bowles EJA, Walker RL, Anderson ML, Dublin S, Crane PK, Larson EB (2017). Risk of Alzheimer’s disease or dementia following a cancer diagnosis. PLoS One.

[8] Khosrow-Khavar F, Rej S, Yin H, Aprikian A, Azoulay L (2017). Androgen deprivation therapy and the risk of dementia in patients with prostate Cancer. J Clin Oncol.

[9] Kang J, Shin DW, Han K, Park SH, Lee WG, Yoo JE (2020). Risk of dementia in prostate cancer survivors: a nationwide cohort study in Korea. Curr Probl Cancer.

[10] Choi YJ, Shin DW, Jang W, Lee DH, Jeong S-M, Park S (2019). Risk of dementia in gastric Cancer survivors who underwent gastrectomy: a Nationwide study in Korea. Ann Surg Oncol.

[11] Schmidt SA, Ording AG, Horvath-Puho E, Sørensen HT, Henderson VW (2017). Non-melanoma skin cancer and risk of Alzheimer's disease and all-cause dementia. PLoS One.

[12] Hopkinson JB, Milton R, King A, Edwards D. People with dementia: what is known about their experience of cancer treatment and cancer treatment outcomes? A systematic review. Psychooncology. 2016. 10.1002/pon.4185. 10.1002/pon.4185 27246507

[13] Cavers D, Habets L, Cunningham-Burley S, Watson E, Banks E, Campbell C (2019). Living with and beyond cancer with comorbid illness: a qualitative systematic review and evidence synthesis. J Cancer Surviv.

[14] Gao W, Gulliford M, Morgan M, Higginson IJ. Primary care service use by end-of-life cancer patients: a nationwide population-based cohort study in the United Kingdom. BMC Fam Pract. 2020;21(1):76. 10.1186/s12875-020-01127-8. 10.1186/s12875-020-01127-8 PMC7191808 32349696

[15] Puts MTE, Monette J, Girre V, Wolfson C, Monette M, Batist G (2010). Does frailty predict hospitalization, emergency department visits, and visits to the general practitioner in older newly-diagnosed cancer patients? Results of a prospective pilot study. Crit Rev Oncol Hematol.

[16] Clague F, Mercer SW, McLean G, Reynish E, Guthrie B (2017). Comorbidity and polypharmacy in people with dementia: insights from a large, population-based cross-sectional analysis of primary care data. Age Ageing.

[17] Browne J, Edwards DA, Rhodes KM, Brimicombe DJ, Payne RA (2017). Association of comorbidity and health service usage among patients with dementia in the UK: a population-based study. BMJ Open.

[18] Bunn F, Burn A-M, Goodman C, Rait G, Norton S, Robinson L (2014). Comorbidity and dementia: a scoping review of the literature. BMC Med.

[19] Ashley L, Kelley R, Griffiths A, Cowdell F, Henry A, Inman H (2021). Understanding and identifying ways to improve hospital-based cancer care and treatment for people with dementia: an ethnographic study. Age Ageing.

[20] Kedia SK, Chavan PP, Boop SE, Yu X (2017). Health care utilization among elderly Medicare beneficiaries with coexisting dementia and Cancer. Gerontol Geriatr Med.

[21] Bradley SH, Lawrence NR, Carder P (2018). Using primary care data for health research in England – an overview. Future Heathcare J.

[22] Wilkinson T, Schnier C, Bush K, Rannikmäe K, Henshall DE, Lerpiniere C (2019). Identifying dementia outcomes in UK biobank: a validation study of primary care, hospital admissions and mortality data. Eur J Epidemiol.

[23] Sutcliffe D, Lester H, Hutton J, Stokes T (2012). NICE and the quality and outcomes framework (QOF) 2009-2011. Qual Prim Care.

[24] SystmOne. https://tpp-uk.com/products/. Accessed 18 Oct 2022.

[25] ResearchOne. ResearchOne, http://www.researchone.org/ Accessed 19 Mar 2021, (n.d.).

[26] Office for National Statistics (2019). Population estimates for the UK, England and Wales, Scotland and Northern Ireland: mid-2018.

[27] NHS Digital (2020). Read Codes.

[28] Cancer Research UK (2017). Cancer incidence for common cancers.

[29] Prince M, Knapp M, Guerchet M, McCrone P, Prina M, Comas-Herrera A (2014). Dementia UK: update.

[30] National Cancer Institute (2019). Understanding cancer prognosis.

[31] Office for National Statistics (2015). Harmonised conepts and questions for social data sources. Primary principles: ethnic group.

[32] Department for Communities and Local Government (2015). The English Index of Multiple Deprivation (IMD) 2015 – Guidance.

[33] Office for National Statistics (2017). Output Area to Region (December 2017) Lookup in England and Wales.

[34] Office for National Statistics (2011). National statistics postcode look-up user guide.

[35] Yirga AA, Melesse SF, Mwambi HG, Ayele DG (2020). Negative binomial mixed models for analyzing longitudinal CD4 count data. Sci Rep.

[36] SAS Institute Inc (2016). SAS® 9.4.

[37] Donegan K, Fox N, Black N, Livingston G, Banerjee S, Burns A (2017). Trends in diagnosis and treatment for people with dementia in the UK from 2005 to 2015: a longitudinal retrospective cohort study. Lancet Public Health.

[38] Alzheimer’s Research UK (2018). Dementia diagnosis rate.

[39] Farrington N, Richardson A, Bridges J. Interventions for older people having cancer treatment: a scoping review. J Geriatr Oncol. 2020. 10.1016/j.jgo.2019.09.015. 10.1016/j.jgo.2019.09.015 31699674

[40] Sarfati D, Koczwara B, Jackson C (2016). The impact of comorbidity on cancer and its treatment. CA Cancer J Clin.

[41] Livingston G, Huntley J, Sommerlad A, Ames D, Ballard C, Banerjee S (2020). Dementia prevention, intervention, and care: 2020 report of the lancet commission. Lancet.

[42] Sleeman KE, Perera G, Stewart R, Higginson IJ (2018). Predictors of emergency department attendance by people with dementia in their last year of life: retrospective cohort study using linked clinical and administrative data. Alzheimers Dement.

[43] Roy M, Halbert B, Devlin S, Chiu D, Graue R, Zerillo JA. From metrics to practice: identifying preventable emergency department visits for patients with cancer. Support Care Cancer. 2020. 10.1007/s00520-020-05874-3. 10.1007/s00520-020-05874-3 33159604

[44] Lash RS, Bell JF, Reed SC, Poghosyan H, Rodgers J, Kim KK (2017). A systematic review of emergency department use among Cancer patients. Cancer Nurs.

[45] LaMantia MA, Stump TE, Messina FC, Miller DK, Callahan CM (2016). Emergency department use among older adults with dementia. Alzheimer Dis Assoc Disord.

[46] Shepherd H, Livingston G, Chan J, Sommerlad A (2019). Hospitalisation rates and predictors in people with dementia: a systematic review and meta-analysis. BMC Med.

[47] Blytt KM, Selbæk G, Drageset J, Natvig GK, Husebo BS (2018). Comorbid dementia and Cancer in residents of nursing homes: secondary analyses of a cross-sectional study. Cancer Nurs.

[48] Liu S-H, Hunnicutt JN, Ulbricht CM, Dubé CE, Hume AL, Lapane KL (2019). Adjuvant use and the intensification of pharmacologic Management for Pain in nursing home residents with Cancer: data from a US National Database. Drugs Aging.

[49] Boyd M, Frey R, Balmer D, Robinson J, McLeod H, Foster S (2019). End of life care for long-term care residents with dementia, chronic illness and cancer: prospective staff survey. BMC Geriatr.

[50] Geramita EM, Parker IR, Brufsky JW, Diergaarde B, van Londen GJ (2020). Primary care providers’ knowledge, attitudes, beliefs, and practices regarding their preparedness to provide Cancer survivorship care. J Cancer Educ.

[51] McDonough AL, Rabin J, Horick N, Lei Y, Chinn G, Campbell EG (2019). Practice, preferences, and practical tips from primary care physicians to improve the Care of Cancer Survivors. J Oncol Pract.

[52] Mansfield E, Noble N, Sanson-Fisher R, Mazza D, Bryant J (2019). Primary care physicians’ perceived barriers to optimal dementia care: a systematic review. The Gerontologist.

[53] Lee L, Hillier LM, Patel T, Weston WW (2020). A decade of dementia care training: learning needs of primary care clinicians. J Contin Educ Heal Prof.

[54] Lisy K, Kent J, Piper A, Jefford M (2021). Facilitators and barriers to shared primary and specialist cancer care: a systematic review. Support Care Cancer.

