# Supplementary material for: Characteristics and general practice resource use of people with comorbid cancer and dementia in England: a retrospective cross-sectional study
Source: BMC Prim Care. 2022 Nov 12;23:281. doi: 10.1186/s12875-022-01882-w (PMC9655793; doi:10.1186/s12875-022-01882-w)
Supplement: Supplementary file 1 — Additional file 1: Table S1. Data categories. [file 12875_2022_1882_MOESM1_ESM.docx]

**Table S1: Data categories**

| **Cancer types** | **Dementia types** | **Ethnicity categories** | **Geographical Region** | **Long-term conditions** |
| --- | --- | --- | --- | --- |
| Breast  Prostate  Lung  Bowel  Melanoma skin cancer  Non-Hodgkin Lymphoma  Kidney  Head and neck  Brain, other Central Nervous System and intracranial tumours  Pancreas  Bladder  Leukaemia  Uterus  Oesophagus  Cancer of unknown primary  Ovary  Stomach  Liver  Myeloma  Thyroid  Other | Alzheimer’s disease  Vascular dementia  Mixed dementia  Dementia with Lewy bodies  Frontotemporal dementia  Parkinson’s dementia  Other | White  Black/African/Caribbean/Black British  Asian/Asian British  Mixed/Multiple ethnic groups  Other ethnic group  Not stated | East Midlands  East of England  London  North East  North West  South East  South West  West Midlands  Yorkshire and the Humber  Isle of Man  Channel Islands | Atrial Fibrillation  Anxiety  Asthma  Chronic Kidney Disease  Chronic Obstructive Pulmonary Disease  Chronic Pain  Coronary Heart Disease  Dementia  Depression  Diabetes  Epilepsy  Heart Attack  Heart Failure  Hypertension  Thyroid disorders  Liver Disease  Mental Health / Psychiatric  Osteoarthritis  Osteoporosis  Rheumatism  Stroke |
